# 

**Published:** 1914-09-05

**Authors:** 


### CONTENTS.

| | PAGE |
|---|---|
| **Medicine as a Career** ... ... ... ... ... | 611 |
| **Hospital and Institutional News** ... ... ... | 613 |
| Our Educational Number—The First Wounded to Arrive—A Hospital as a Recruiting Station—Vaccination and the Army—A South African Secretary on Hospital Construction—A Hand-book for Committee-men—The Asylums Board and the Navy——The Outlook for Hospital Income—The War and Medical Qualification—War Accommodation in Salford—Arrival of Wounded at Chatham—Emergency Call at Gravesend—School Medical Officers on Active Service—Local Government Board Advice on Drug Contracts—Infirmaries and Schemes of Work—The Bolton Guardians' New Buildings—A Gold Medal for Army Sanitation—The Manufacture of Foreign Drugs—The Navy and the Drug Market. | |
| **Hospitals and the War** ... ... ... ... | 617 |
| The Provincial Hospitals' Arrangements (From Our Special Correspondents) : Bath—Bradford—Leeds — Leicester — Lincoln — North Devon Division—Sheffield. | |
| **War Patients at the Hospital** ... ... ... | 619 |
| **The Royal Naval Medical Service** ... ... | 621 |
| What it Offers to the Newly Qualified Medical Man. | |
| **The Study of Tropical Medicine** ... ... ... | 622 |
| In London, Liverpool, and Edinburgh. | |

### CONTENTS—(*continued*).

PAGE

**The Royal Army Medical Corps** ... ... ... 623

A Review of the Prospects and Pay.

**Life in the Indian Medical Service** ... ... 626
The Attractions of Medicine and Sport.

**The Medical Curriculum** ... ... ... ... 627
Qualification and Registration.
The English Conjoint Course.
The Fellowship of the Royal College of Surgeons.
The Cambridge University Course.
Oxford University.
The London University Course.
Higher Diplomas.

**Institutional Needs** ... ... ... ... ... 640

PAGE

**The Medical Student and his Work** ... ... 630
The Medical Schools of the United Kingdom: London — Provincial Schools — Scotland—Ireland.

**The Public Services** ... ... ... ... ... 636
Royal Naval Medical Service—The Royal Army Medical Corps—The Indian Medical Service—The Colonial Medical Service.

**London Post-Graduate Institutions** ... ... 638
West London Post-Graduate College—The London Polyclinic—The London School of Clinical Medicine (Post-Graduate)—North-East London Post-Graduate College.

**Opportunities for Graduate Study in Special Hospitals** ... ... ... ... ... ... 639

**The Poor-Law Service and the War** ... ... 640

